# The Efficacy of Mizoribine (Inosine Monophosphate Dehydrogenase Inhibitor) for ANCA-Associated Vasculitis with Hepatitis B Virus Carrier

**DOI:** 10.1155/2012/929318

**Published:** 2012-12-11

**Authors:** Jun Muratsu, Atsuyuki Morishima, Masayoshi Kukida, Anzu Tanaka, Shigeki Fujita, Katsuhiko Sakaguchi

**Affiliations:** ^1^Department of Nephrology and Hypertension, Sumitomo Hospital, 5-3-20 Kitaku Nakanoshima, Osaka 530-0005, Japan; ^2^Department of Pathology, Sumitomo Hospital, 5-3-20 Kitaku Nakanoshima, Osaka 530-0005, Japan

## Abstract

A 42-year-old female who was an asymptomatic carrier of hepatitis B virus (HBV) was diagnosed with antineutrophil cytoplasm antibody- (ANCA-) associated vasculitis and was induced to remission with 30 mg/day prednisolone nine years ago. Four years ago, she suffered recurrence of ANCA-associated vasculitis and with 30 mg/day prednisolone was induced to remission. This time, laboratory data showed 3-fold increase in myeloperoxidase antineutrophil cytoplasmic antibody (MPO-ANCA) levels. Administration of 30 mg/day prednisolone was started. Three days later, she was admitted to our hospital suffering from fatigue. After admission, urinalysis showed glomerular hematuria. Despite administration of 30 mg/day prednisolone, MPO-ANCA titer had been of high level, ranging from 42 to 83 EU for 2.5 months. Furthermore, the adverse effects of steroid were seen. We decided the tapering of prednisolone (25 mg/day) and the start of mizoribine (4-carbamoyl-1-*β*-D-ribofuranosyl imidazolium-5-olate) administration. After mizoribine treatment, MPO-ANCA titer was decreased without any mizoribine-related adverse effects. Six months later, MPO-ANCA titer was decreased to normal levels and she was induced to clinical remission without reactivation of HBV. We describe the effectiveness of mizoribine for the ANCA-associated vasculitis complicated with HBV-carrier.

## 1. Introduction

The common treatment for antineutrophil cytoplasm antibody- (ANCA-) associated vasculitis is oral cyclophosphamide-corticosteroid combination therapy. However, there are some reports that cyclophosphamide-corticosteroid combination therapy has serious complications such as increased risk of infection, leucopenia, osteoporosis, diabetes, and sterility and malignancy [1–4]. Reactivation of hepatitis B virus (HBV) replication is one of complications in patients with chronic HBV infection who receive immunosuppressive therapy [5]. 

Mizoribine (4-carbamoyl-1-*β*-D-ribofuranosyl imidazololium-5-olate), a purine synthesis inhibitor, has an immunosuppressive effect equivalent to that of azathioprine, but with less hepatic toxicity and myelosuppression [6]. Mizoribine and mycophenolic acid inhibit the rate-limiting enzyme inosine monophosphate dehydrogenase (IMPDH) in the *de novo* pathway of purine biosynthesis. It was reported that IMPDH inhibitors have potential antiviral effect *in vitro* and inhibited HBV replication with cultures of primary human hepatocytes, HepG2 2.2.15 cells [7–12].

There have been some reports that mizoribine is useful not only as a preemptive treatment to prevent relapse, but as also an aggressive strategy to induce the remission of relapsed ANCA-associated renal vasculitis [13, 14]. However, there have not been any reports about the effectiveness of mizoribine in ANCA-associated renal vasculitis with HBV carrier. Herein, we describe the successful treatment with mizoribine for the case of the ANCA-associated renal vasculitis complicated with HBV carrier who was induced to remission without reactivation of HBV. 

## 2. Case Presentation

**Figure 1:**
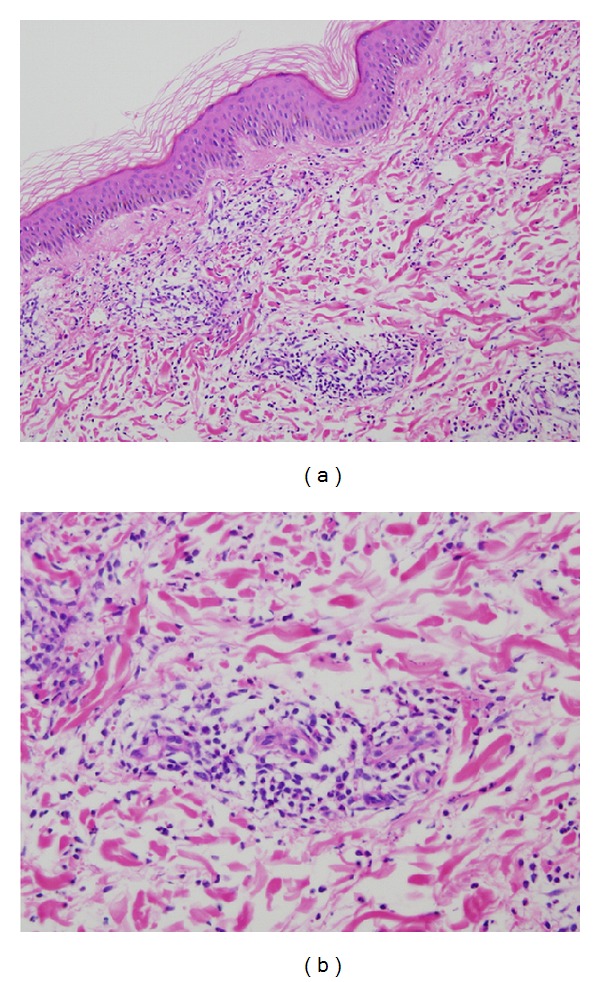
Skin biopsy showed findings of leukocytoclastic vasculitis (A, B H&E stain; A × 20; B × 40).

A 42-year-old Japanese female experienced glomerular hematuria, bilateral pedal purpura, and arthralgia nine years ago. Elevation of MPO-ANCA titer and serum creatinine levels was shown. Percutaneous renal biopsy was performed. Nine glomeruli were observed by light microscopy. Five of them were totally obsolescent, and the rest were also collapsing to some extent. Two glomerulus showed cellular crescentic glomerulonephritis. Glomerular deposition of IgA, IgM, IgG, C1q, C3, and C4 was negative. In addition, skin biopsy was performed, which showed leukocytoclastic vasculitis (Figures 1(a) and 1(b)). Thus, she was diagnosed with antineutrophil cytoplasm antibody- (ANCA-) associated vasculitis. Her past medical histories included asymptomatic carrier of hepatitis B virus (HBV) in childhood. Administration of lamivudine was started. She was induced to remission with 30 mg/day prednisolone. Prednisolone had been gradually tapered to 5 mg. Elevation of aspartateaminotransferase and alanine aminotransferase had not been seen. Four years ago, the ANCA-associated vasculitis relapsed and was induced to remission 30 mg/day prednisolone. Prednisolone had been gradually tapered to 12 mg. HBV-DNA had been from 3.4 to 4.5log(10)IU. Elevation of aspartateaminotransferase and alanine aminotransferase was not seen. 

**Figure 2:**
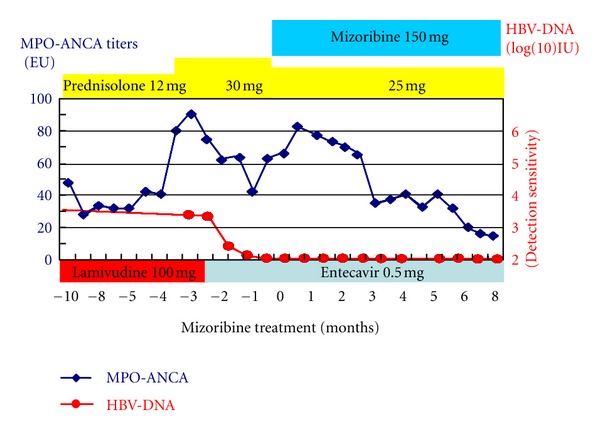
Time course plots of the MPO-ANCA levels and HBV-DNA. *MPO-ANCA*: myeloperoxidase antineutrophil cytoplasmic antibody; *HBV-DNA*: Hepatitis B Virus-DNA.

**Figure 3:**
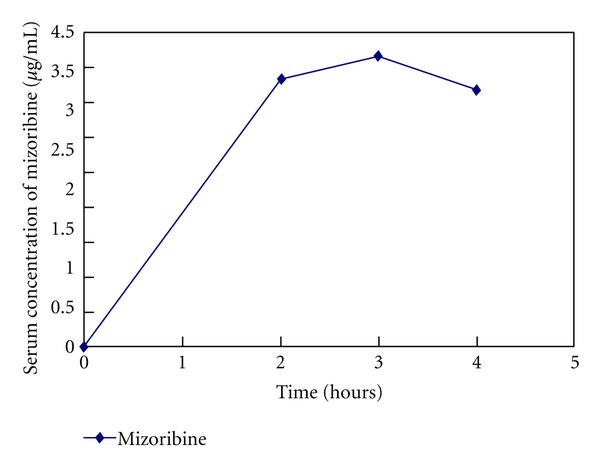
Serum concentration-time curve of mizoribine in this case.

This time, laboratory data showed normal serum creatinine levels and, however, 3-fold increase in myeloperoxidase antineutrophil cytoplasmic antibody (MPO-ANCA) levels (82 EU). We speculated recurrence of the ANCA-associated vasculitis. Administration of 30 mg/day prednisolone was started. Three days later, she was admitted to our hospital suffering from fatigue. On admission, her pulse rate was 76/min regular, blood pressure was 130/84 mmHg, and body temperature was 36.2°C. Her consciousness was clear. Her bulbar conjunctiva was not icteric, and the palpebral conjunctiva was not pale. On auscultation, her lungs and heart were normal. Cough, pulmonary alveolar hemorrhage, and gastrointestinal bleeding were not observed. On palpation, hepatosplenomegaly and ascites were not observed. She did not have any neurological abnormalities. In addition, purpura, rash, arthralgia, myalgia, edema, or oral and genital ulceration were not found.  Table 1 shows laboratory data at the admission. Laboratory data showed myeloperoxidase antineutrophil cytoplasmic antibody (MPO-ANCA) levels and showed 3-fold increase, 93 EU. HBs antigen was positive, and HBV-DNA level was 3.4log(10)IU. Her chest X-ray and echocardiography were normal and electrocardiography had no problem. Abdominal ultrasonography showed that liver was not atrophy. After admission, a urinalysis indicated +/−  proteinuria and a 3+ occult blood reaction with 21–50 red blood cells per high-power field. Her 24-hour urinary protein excretion was 0.03 g. Her urine volume was 2300 mL/day.  Figure 2 shows time course plots of the MPO-ANCA levels and HBV-DNA. Despite administration of 30 mg/day prednisolone for 2.5 months, MPO-ANCA titers increased and hematuria had been seen without elevation of serum creatinine levels. Furthermore, the adverse effects of steroid, such as moon face, insomnia, and femur head necrosis, were seen. Because she strongly desired pregnancy in the future, we did not start cyclophosphamide. We decided tapering of prednisolone (25 mg/day) and start of mizoribine (4-carbamoyl-1-*β*-D-ribofuranosyl imidazololium-5-olate) (150 mg/day) administration. Before the start of mizoribine, HBV-DNA was 2.5log(10)IU and elevation of aspartateaminotransferase and alanine aminotransferase was not seen. Because HBV-DNA was detectable and longer duration (>12 months) of treatment for HBV and ANCA-associated renal vasculitis is anticipated, we changed lamivudine to entecavir before the start of mizoribine administration. We monitored serum concentration of mizoribine (Figure 3). Trough level of mizoribine was 0 *μ*g/mL, after 2 hours the level was 3.82 *μ*g/mL, after 3 hours the level (peak level of mizoribine) was 4.17 *μ*g/mL, and after 4 hours the level was 3.67 *μ*g/mL. After mizoribine treatment, MPO-ANCA titer was decreased without any mizoribine-related adverse effects. Six months later, hematuria and fatigue had not been shown. She was induced to clinical remission without reactivation of HBV. Elevation of MPO-ANCA titers has not been presented for one year. 

## 3. Discussion

We describe a case of the ANCA-associated vasculitis with HBV carrier who was induced remission with mizoribine without reactivation of HBV.

In general, the treatment of relapsed ANCA-associated vasculitis is based on cyclophosphamide and high-dose corticosteroids. However, high-dose corticosteroids are of high risk for HBV replication because HBV-DNA contains a glucocorticoid responsive element [15]. Reactivation of hepatitis B virus (HBV) replication is a well-recognized complication in patients with chronic HBV infection who receive immunosuppressive therapy [5]. However, it was reported that corticosteroid is relatively safe under administration of entecavir [16]. No reports have been described if mizoribine caused HBV replication and fulminant hepatitis. 

Mizoribine has an immunosuppressive effect equivalent to that of azathioprine; however, it shows less hepatic toxicity and myelosuppression [6]. Mizoribine has recently been proved clinically effective and relatively safe for the treatment of nephritic syndrome, lupus nephritis, IgA nephropathy, and ANCA-associated renal vasculitis [14, 17–19]. The administration should also only be done in facilities where the blood level of mizoribine can be monitored. It is important to monitor serum concentration of mizoribine because mizoribine is excreted through kidney. There have been some reports that more than 5 *μ*g/mL of mizoribine trough level induce hepatic dysfunction [6]. Mizoribine showed an inhibition of 50% on human mixed-lymphocyte reaction at a concentration of about 1 *μ*g/mL. 

Mizoribine and mycophenolic acid inhibit the rate-limiting enzyme inosine monophosphate dehydrogenase (IMPDH) in the de novo pathway of purine biosynthesis. It has been reported that mycophenolic acid inhibits the replication of human immunodeficiency virus (HIV) *in vitro* by depletion of the deoxynucleoside triphosphate substrate of the reverse transcriptase, thus blocking formation of the viral DNA [7]. It was reported that *in vitro* mycophenolic acid inhibited HBV replication. Experiments were performed using cultures of primary human hepatocytes, HepG2 2.2.15 cells in the report [8]. Inhibition of IMPDH reduces the level of intracellular guanine nucleotides required for adequate RNA and DNA synthesis. Therefore, IMPDH inhibitors have potential antiviral effect [9–12]. In addition, it was reported that mizoribine was able to suppress cytomegalovirus plaque formation dose dependently [20]. Thus, we speculate that mizoribine would inhibit HBV replication. However, there was no evidence that mizoribine inhibited HBV replication *in vivo*. Entecavir may suppress HBV replication in this case. This case revealed that mizoribine is safe for ANCA-associated renal vasculitis complicated with HBV carrier under administration of entecavir. It is important to consider mizoribine administration for ANCA-associated renal vasculitis with HBV carrier. However, we must take care of reactivation of HBV replication and monitor HBV-DNA. 

In conclusion, mizoribine may be useful and safe for ANCA-associated vasculitis with HBV carrier. There is the possibility that mizoribine is one of the choices for the treatment of ANCA-associated renal vasculitis complicated with HBV carrier. Further experience is needed to confirm our conclusions. 

## Figures and Tables

**Table 1:** Laboratory data on admission.

*Complete blood count *	
White blood cell count	12100/*μ*L
Red blood cell count	354 × 10^4^/*μ*L
Hemoglobin	11.9 g/dL
Hematocrit	35.8%
Platelets	33.1 × 10^4^/*μ*L
*Blood chemical *	
Aspartateaminotransferase	23 IU/L
Alanine aminotransferase	12 IU/L
Alkaline phosphatase	117 IU/L
Lactate dehydrogenase	202 IU/L
*γ*-GTP	24 IU/L
Total bilirubin	0.7 mg/dL
Creatine phosphokinase	77 IU/L
Total cholesterol	312 mg/dL
Triglyceride	69 mg/dL
Sodium	136 mEq/L
Chloride	99 mEq/L
Potassium	4.5 mEq/L
Uric acid	4.2 mg/dL
Blood urea nitrogen	13 mg/dL
Creatinine	0.56 mg/dL
Total protein	6.7 g/dL
Albumin	4.6 g/dL
C-reactive protein	0.04 mg/dL
Fasting plasma glucose	79 mg/dL
Hemoglobin A1c	5.0%
KL-6	151 U/mL
*Immunology *	
Immunoglobulin G	782 mg/dL
Immunoglobulin A	145 mg/dL
Immunoglobulin M	48 mg/dL
Complement titer (CH50)	36.9 U/mL
Complement C3	84 mg/dL
Complement C4	18 mg/dL
Antinuclear antibody	<40
HBs antigen	(+)
HBe antigen	(−)
HBe antibody	(+)
HBV-DNA	3.4 log (10) IU
HCV-antibody	(−)
*Coagulation *	
APTT	25.1 second
PT-INR	0.80
<ELISA>	
MPO-ANCA	93 EU
PR3-ANCA	<10 EU
*Urinalysis *	
pH	7.0
Specific gravity	1.010
Glucose	(−)
Protein	(−)
Cast	Hyaline cast (+)
Erythrocytes	1–5/HPF
Leukocytes	1–5/HPF

*γ*-GTP: gamma-glutamyl transpeptidase, PR3-ANCA: proteinase-3 anti-neutrophil cytoplasmic antibody, MPO-ANCA: myeloperoxidase anti-neutrophil cytoplasmic antibody, APTT: activated partial thromboplastin time, PT-INR: prothrombin time-international normalized ratio.
